# Supplementary material for: Arginine-Rich Cell-Penetrating Peptide-Mediated Transduction of Mouse Nasal Cells with FOXP3 Protein Alleviates Allergic Rhinitis
Source: Pharmaceutics. 2023 Jun 19;15(6):1770. doi: 10.3390/pharmaceutics15061770 (PMC10303077; doi:10.3390/pharmaceutics15061770)
Supplement: Supplementary file 1 [file pharmaceutics-15-01770-s001.zip › pharmaceutics-2403941-supplementary.pptx]

## Slide 1
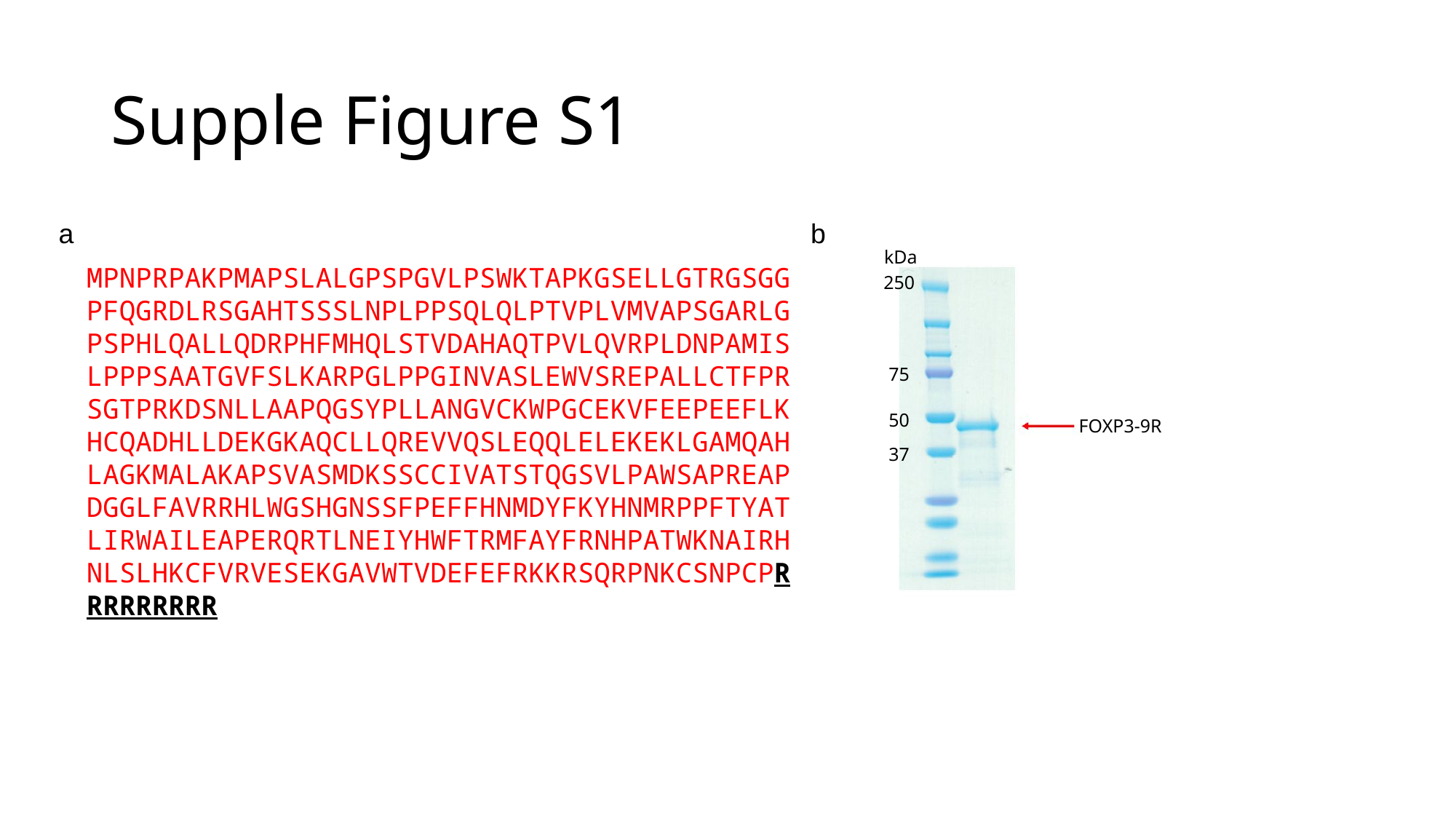

# Supple Figure S1
a
b
kDa
MPNPRPAKPMAPSLALGPSPGVLPSWKTAPKGSELLGTRGSGGPFQGRDLRSGAHTSSSLNPLPPSQLQLPTVPLVMVAPSGARLGPSPHLQALLQDRPHFMHQLSTVDAHAQTPVLQVRPLDNPAMISLPPPSAATGVFSLKARPGLPPGINVASLEWVSREPALLCTFPRSGTPRKDSNLLAAPQGSYPLLANGVCKWPGCEKVFEEPEEFLKHCQADHLLDEKGKAQCLLQREVVQSLEQQLELEKEKLGAMQAHLAGKMALAKAPSVASMDKSSCCIVATSTQGSVLPAWSAPREAPDGGLFAVRRHLWGSHGNSSFPEFFHNMDYFKYHNMRPPFTYATLIRWAILEAPERQRTLNEIYHWFTRMFAYFRNHPATWKNAIRHNLSLHKCFVRVESEKGAVWTVDEFEFRKKRSQRPNKCSNPCPRRRRRRRRR
250
75
50
FOXP3-9R
37

## Slide 2
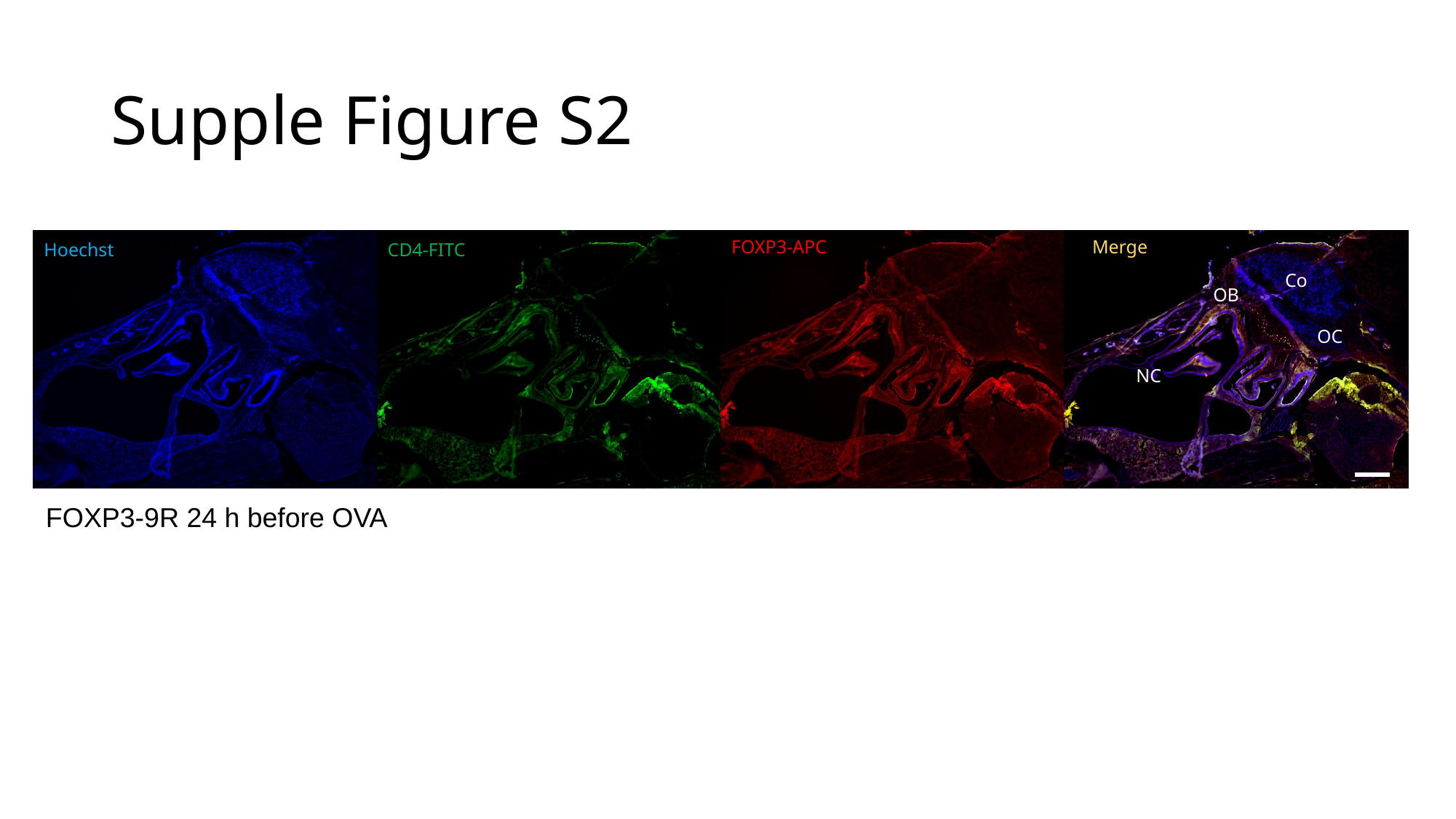

# Supple Figure S2
FOXP3-APC
Merge
Hoechst
CD4-FITC
Co
OB
OC
NC
FOXP3-9R 24 h before OVA

## Slide 3
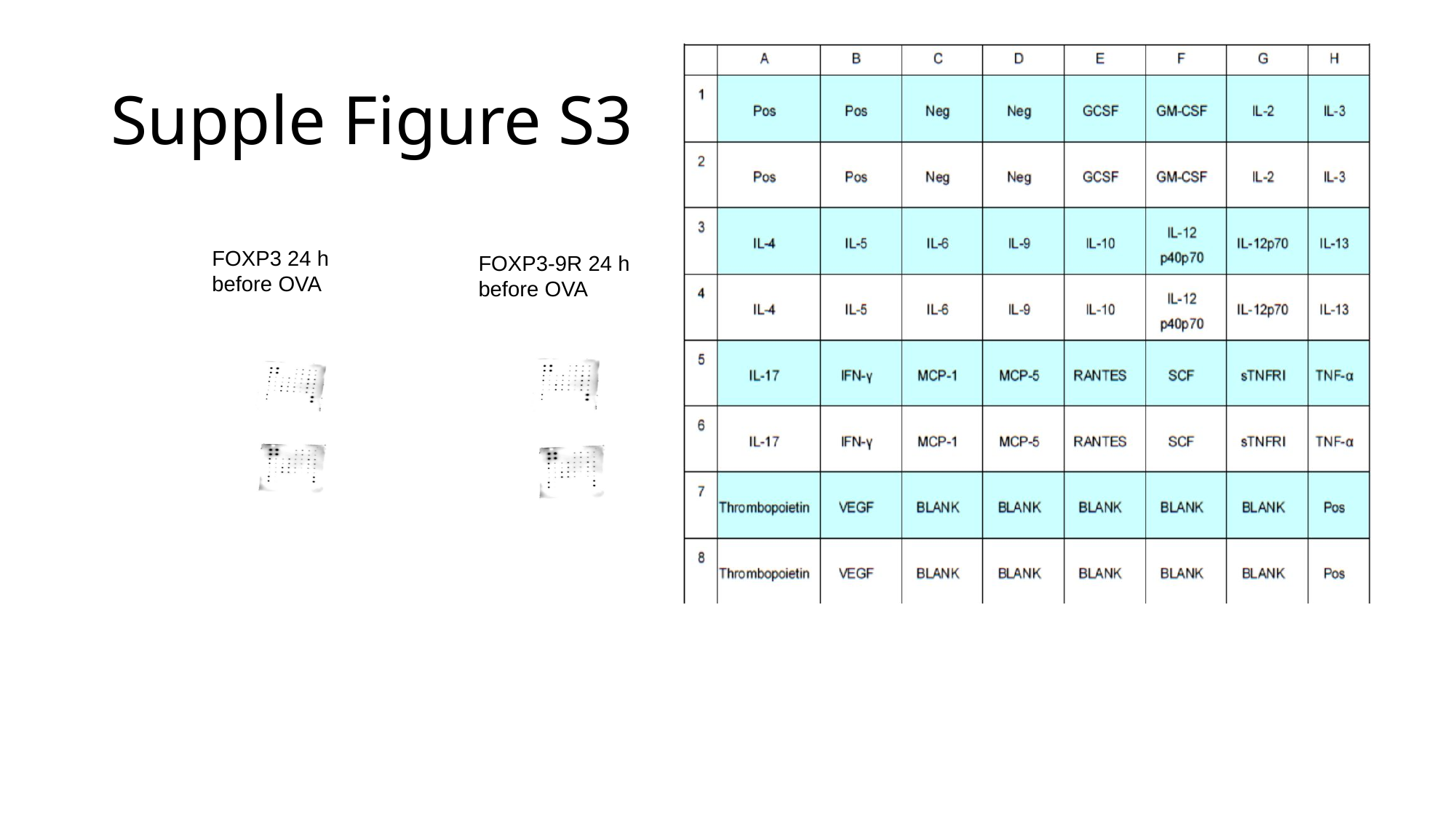

# Supple Figure S3
FOXP3 24 h
before OVA
FOXP3-9R 24 h
before OVA

## Slide 4
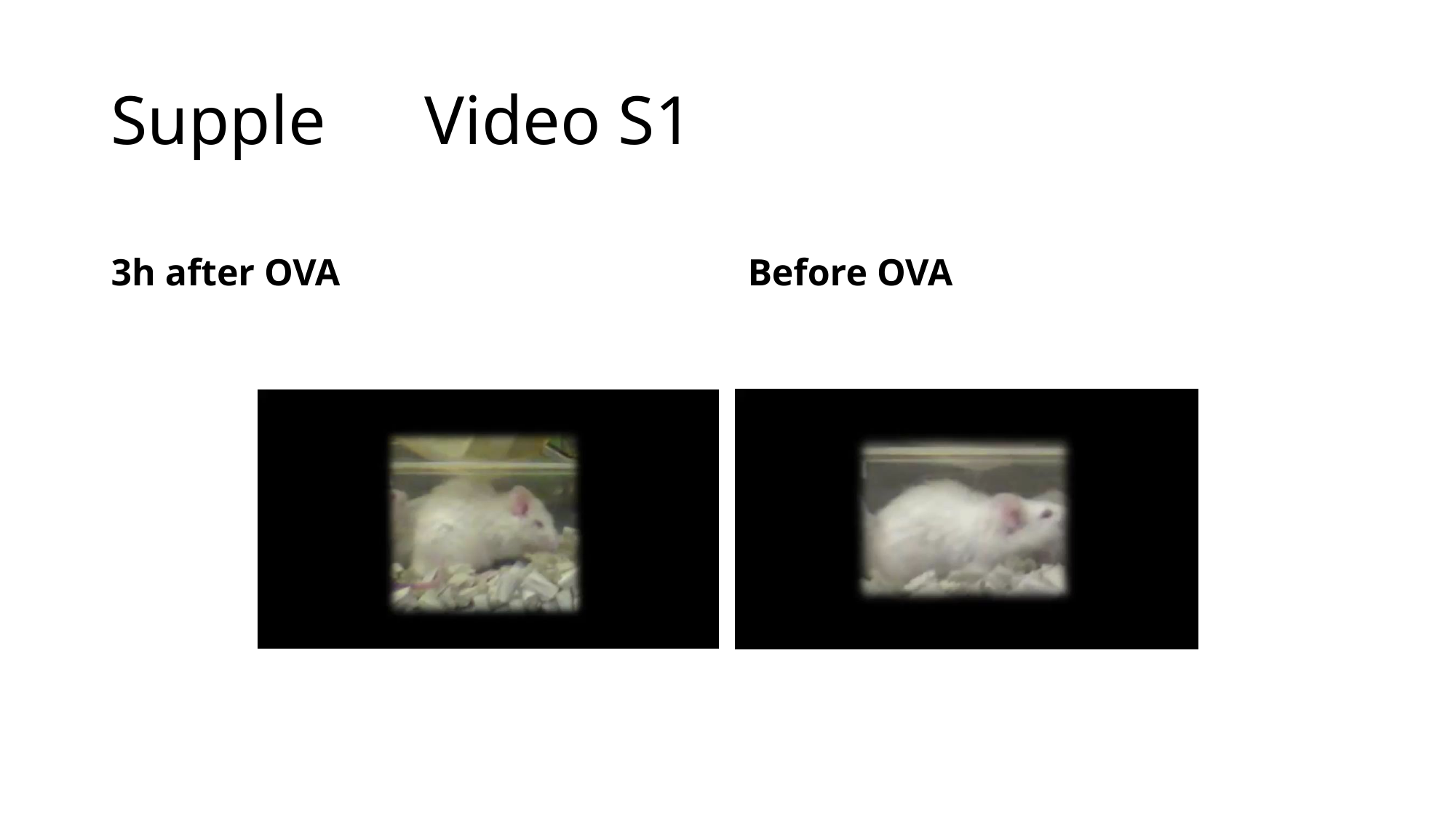

# Supple　Video S1
3h after OVA
Before OVA
